# The Protective Effects of Maresin 1 in the OVA-Induced Asthma Mouse Model

**DOI:** 10.1155/2021/4131420

**Published:** 2021-02-10

**Authors:** Guochun Ou, Qin Liu, Chengxiu Yu, Xiaoju Chen, Wenbo Zhang, Yong Chen, Tao Wang, Yongqiang Luo, Guolu Jiang, Mingmei Zhu, Hongmei Li, Mei Zeng

**Affiliations:** ^1^Department of Respiratory and Critical Care Medicine, The Affiliated Hospital of North Sichuan Medical College, Nanchong, Sichuan 637000, China; ^2^Department of Respiratory and Critical Care Medicine, Suining Central Hospital, Suining, Sichuan 629000, China; ^3^Department of Geriatric Medicine, Chengdu Fifth People's Hospital, Chengdu, Sichuan 611130, China; ^4^Department of Gastroenterology, Anyue County People's Hospital, Ziyang, Sichuan 642350, China; ^5^Department of Transfusion, Suining Central Hospital, Suining, Sichuan 629000, China; ^6^Institute of Rheumatology and Immunology, The Affiliated Hospital of North Sichuan Medical College, Nanchong, Sichuan 637000, China

## Abstract

Asthma is a chronic inflammatory disease that cannot be cured. Maresin 1 (MaR1) is a specific lipid synthesized by macrophages that exhibits powerful anti-inflammatory effects in various inflammatory diseases. The goal of this study was to evaluate the effect of MaR1 on allergic asthma using an ovalbumin- (OVA-) induced asthma model. Thirty BALB/c mice were randomly allocated to control, OVA, and MaR1 + OVA groups. Mice were sacrificed 24 hours after the end of the last challenge, and serum, bronchoalveolar lavage fluid (BALF), and lung tissue were collected for further analysis. Western blotting was used to measure the protein level of I*κ*B*α*, the activation of the NF-*κ*B signaling pathway, and the expression of NF-*κ*B downstream inflammatory cytokines. Quantitative real-time polymerase chain reactions (qRT-PCRs) were used to evaluate the expression levels of COX-2 and ICAM-1 in lung tissues. We found that high doses of MaR1 were most effective in preventing OVA-induced inflammatory cell infiltration and excessive mucus production in lung tissue, reducing the number of inflammatory cells in the BALF and inhibiting the expression of serum or BALF-associated inflammatory factors. Furthermore, high-dose MaR1 treatment markedly suppressed the activation of the NF-*κ*B signaling pathway, the degradation of I*κ*B*α*, and the expression of inflammatory genes downstream of NF-*κ*B, such as COX-2 and ICAM-1, in the OVA-induced asthma mouse model. Our findings indicate that MaR1 may play a critical role in OVA-induced asthma and may be therapeutically useful for the management of asthma.

## 1. Introduction

Asthma is an allergic disease affecting 300 million people worldwide [1]. It is a chronic respiratory disease distinguished by bronchial hyperresponsiveness, airflow obstruction, and airway inflammation [2]. Glucocorticoids have become the first choice for the treatment of asthma due to their anti-inflammatory effect [3], but long-term use can cause obvious and even irreversible side effects [4]. Furthermore, approximately 5%-10% of asthma patients are not sensitive to hormones and have glucocorticoid resistance [5]. Therefore, these challenges highlight the need for the development of alternate strategies for asthma control.

NF-*κ*B is the core mediator of the inflammatory response [6]. Normally, NF-*κ*B is inhibited by I*κ*B*α* and in a resting state; however, activation of the receptor system of inflammation causes I*κ*B*α* degradation, thereby activating NF-*κ*B [7]. Several studies have shown that prolonged activation of NF-*κ*B is linked to the development of asthma [8, 9]. Under pathological conditions, NF-*κ*B regulates the expression of ICAM-1 in vascular endothelial cells and lung epithelial cells, and studies have shown that NF-*κ*B can promote the differentiation of Th2 type cells and increase the levels of proinflammatory cytokines [10]. In inflammatory airway epithelial cells, upregulation of ICAM-1 causes increased adhesion of eosinophils to endothelial cells [11]. COX-2 is mainly present in airway epithelial cells and smooth muscle cells and is minimally expressed under physiological conditions [12]. In patients with asthma, there is a high number of inflammatory cells infiltrating the airway mucosa, which can generate a large amount of COX-2 after activation [13]. COX-2 can also generate a large number of inflammatory mediators, which in turn aggravate the inflammatory response, thus inducing or aggravating asthma [14]. Recently, Kostyra demonstrated that osthole has potential for the treatment of allergies via the inhibition of the COX-2 pathway [15]. As noted above, NF-*κ*B, ICAM-1, and COX-2 are involved in allergic asthma.

Maresin 1 (MaR1), a lipoprotein derived from *ω*-3 unsaturated fatty acids, is one of the newest families of mediators promoting inflammatory regression [16]. Among the specialized proresolving lipid mediators (SPMs) is MaR1, which has inhibitory effects in experimental inflammatory disease models, including rheumatoid arthritis, colitis, acute lung injury, and nonalcoholic steatohepatitis [17–20]. Previous studies have reported that MaR1 plays a protective role in various chronic inflammatory diseases by inhibiting neutrophil infiltration, reducing the production of proinflammatory factors, enhancing macrophage phagocytosis, and inhibiting NF-*κ*B activation [21]. The purpose of this study was to examine whether MaR1 has an ameliorative effect on OVA-induced asthma in a mouse model and to further investigate the possible molecular mechanisms.

## 2. Materials and Methods

### 2.1. Animals and Experimental Protocol

Prior to commencing the study, ethical clearance was sought from the Ethics Committee of the North Sichuan Medical College, and all mouse experiments were carried out in accordance with the requirements of the Institutional Animal Care and Use Committee. Thirty female BALB/c mice, 6 weeks old weighing approximately 20 g, and free of murine-specific pathogens were obtained from North Sichuan Medical College (Nanchong, Sichuan, China). According to the requirements of the research, the animals were randomly assigned to five groups (*n* = 6): the control group, OVA group, and MaR1 (low dose (0.1 ng), medium dose (1 ng), or high dose (10 ng)/per mouse) + OVA groups. In the breeding environment, mice were given standard laboratory chow and water and were maintained under controlled conditions of 12 : 12-hour light-dark cycle at 22-24°C.

### 2.2. Sensitization and Induction of Airway Inflammation with OVA

**Figure 1:**
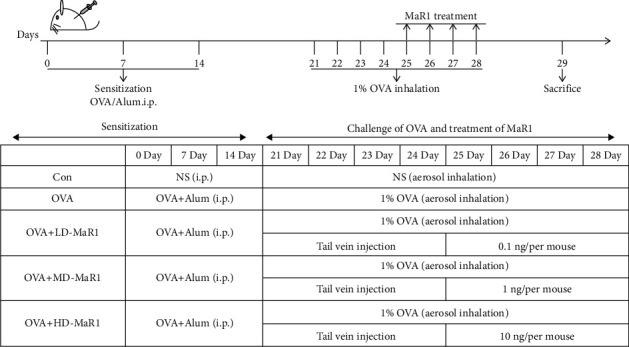
Mouse model of allergic airway inflammation, and treatment with MaR1.

The experimental schedule was prepared by adapting the procedure used by Liu et al. [22], with some modifications.  Figure 1. shows the establishment of the OVA-induced asthma mouse model and treatment procedure. On days 0, 7, and 14, all mice except the control group were immunized by intraperitoneal injection of a 0.2 ml mixture of 20 *μ*g ovalbumin (grade V, Sigma, St. Louis, MO, USA), 2 mg Imject Alum (Pierce, Rockford, IL, USA), and 0.2 ml saline. From day 21 to day 28, these mice were challenged with ovalbumin (inhalation of 1% ovalbumin) for 30 minutes. In addition, the control mice were treated with 0.9% NaCl. On day 25 to day 28, 20 minutes prior to aerosol challenge, a range of concentrations of MaR1 (Cayman, USA) was injected via the tail vein in the MaR1 + OVA groups; the control group and the OVA model group were given the same volume (0.1 ml/per mouse) of NaCl [23]. Subsequently, after 24 hours, all mice were sacrificed by intraperitoneal injection of 2% sodium pentobarbital (80 mg/kg, Merck, Germany), and BALF and blood and lung tissue were collected for subsequent experiments.

### 2.3. BALF and Differential Cell Counts

After tracheal intubation, the mice were lavaged three times with 1 ml of precooled phosphate-buffered saline (PBS). The gathered BALF was centrifuged at 1200 rpm/min for 10 min at 4°C, and then the supernatants were stored at -80°C for cytokine assays. The cell pellets harvested from BALF were resuspended in 200 *μ*l of PBS. Subsequently, a hemocytometer and the Wright-Giemsa staining was used to assess the total number of white blood cells and the proportion of inflammatory cells (eosinophils, macrophages, neutrophils, and lymphocytes); 200 cells/slide were counted.

### 2.4. Lung Tissue Histopathology

The left lungs were cleaned with PBS, fixed in 4% paraformaldehyde, and dehydrated with an alcohol gradient and transparentized with xylene. After paraffin embedding, the tissues were cut into 5 *μ*m-thick sections, stained with hematoxylin and eosin (H&E; AR1180, Boster, Wuhan, China), and periodic acid–Schiff (PAS) solution (KGA222-1, KeyGen Biotech, Nanjing, China), and examined under an optical microscope. H&E staining was used to observe the pathological changes in the lung tissue, and a score of 0 to 5 was used to evaluate the degree of lung tissue inflammation. PAS staining was used to evaluate the degree of proliferation of airway goblet cells (see the reference for specific ratings [24]).

### 2.5. Enzyme-Linked Immunosorbent Assay

Enzyme-linked immunosorbent assays were used to detect the concentration of inflammatory factors (IL-4, IL-5, and IL-13) in alveolar lavage fluid and IgE in serum. The experimental process was based on the manufacturer's instructions. The reactions were measured at 450 nm by using an ELISA reader, and then the concentration of each inflammatory factor was evaluated according to a standard curve.

### 2.6. Western Blot Assays

Lung samples were homogenized in RIPA buffer (AR0101-100, Boster, Wuhan, China) with a protease inhibitor cocktail. The protein concentration was measured by the BCA method (Thermo Scientific, MA, USA). Samples containing 30 *μ*g of denatured total protein were separated by 10% SDS-PAGE and then transferred to 0.45 *μ*m PVDF membranes. The membranes were blocked with 5% nonfat dry milk for 1 hour and incubated overnight at 4°C with the following antibodies: anti-ICAM-1 (dilution, 1 : 1000, Abcam, Cambridge, Massachusetts, USA), anti-P65 (dilution, 1 : 1000, CST, USA), anti-I*κ*B*α* (dilution, 1 : 2000, HuaBio, Hangzhou, China), anti-p-P65 (dilution, 1 : 1000, CST, USA), anti-COX-2 (dilution, 1 : 1000, HuaBio, Hangzhou, China), and anti-*β*-actin (dilution, 1 : 1000, Bioss, Beijing, China). HRP-conjugated secondary antibodies (dilution, 1 : 5000, BA1054, Boster, Wuhan, China) were incubated for 1 hour at room temperature. Then, proteins were detected by chemiluminescence reagent (AR1171, Boster, Wuhan, China). The gray value of the band was observed and analyzed using the ImageJ software.

### 2.7. qRT-PCR

Fifty milligrams of lung tissue was placed in a grinding bowl, and an appropriate amount of liquid nitrogen was added to immediately grind the lung tissue. Total RNA was extracted with 1 ml of TRIzol (Invitrogen) according to the manufacturer's instructions. Following quantification, 5 *μ*g of mRNA was reverse transcribed to cDNA (Thermo). All-in-one TMMix (Cat. No: QP001, GeneCopoeia, USA) was used as a DNA intercalator dye to quantify DNA amplification. Quantitative PCR was performed on a LightCycler®96 (Roche, Switzerland), and the reaction procedure was 95°C for 10 min, followed by 45 cycles of 95°C for 10 s, 60°C for 20 s, and 72°C for 15 s. *β*-Actin was used as the normalization control. Primer sequences are shown in Table.  1. All the data were calculated with the 2^−*ΔΔ*Ct^ method.

### 2.8. Statistical Analysis

Statistical analyses were performed on the experimental data using SPSS 23.0. The measurement data were expressed as the mean ± SEM. The comparison between the means of multiple groups was performed by one-way ANOVA, the comparisons between two groups were performed by the LSD *t* test. In addition, a *P* value of less than 0.05 was considered statistically significant.

## 3. Results

### 3.1. Pathological Changes in the Lungs of Mice

**Figure 2:**
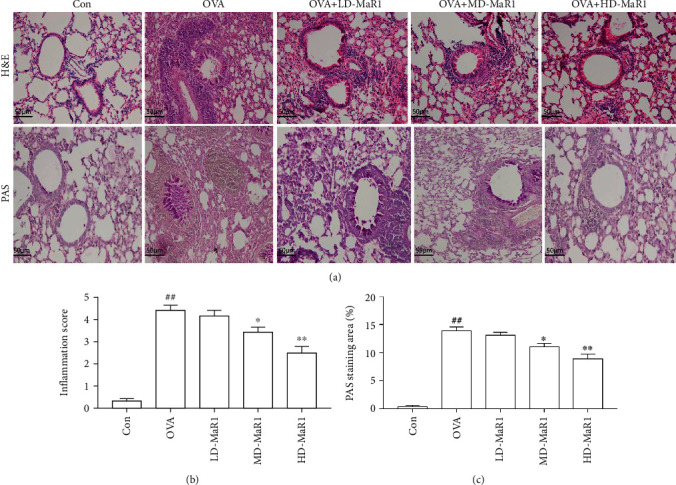
MaR1 treatment suppressed OVA-induced allergic airway inflammation. (a) H&E and PAS staining were used to evaluate inflammatory cell infiltration and mucus production in the lung (×200). (b) Inflammation score. (c) The PAS staining area (%). Data expressed as mean ± SEM (*n* = 6/group). ^##^*P* < 0.01, ^#^*P* < 0.05 vs. the control group; ∗*P* < 0.05, ∗∗*P* < 0.01 vs. OVA.

As shown in Figure 2, PAS and H&E staining (a) were used to evaluate the effect of MaR1 on lung tissue in OVA-induced asthmatic mice. There was a large number of mucus plugs in the bronchial cavity of the mice in the OVA group, the airway epithelial cells were obviously damaged, the goblet cells had proliferated, and there was a large amount of inflammatory cell infiltration around the bronchi and blood vessels, which was in contrast to the histological features of the lungs from the mice in the control group. According to statistical analysis, the inflammation score (b) of the lung tissue from the OVA group and the PAS staining area (c) of the airway were also significantly higher than that in the lung tissue from the control group (all *P* < 0.01). However, MaR1 treatment markedly attenuated OVA-induced lung injury and inflammatory cell infiltration, and the inflammation score and the PAS staining area also decreased (*P* < 0.01 to *P* < 0.05), with high-dose MaR1 group showing the most obvious effect.

### 3.2. Effects of MaR1 on Inflammatory Cell Recruitment in BALF

**Figure 3:**
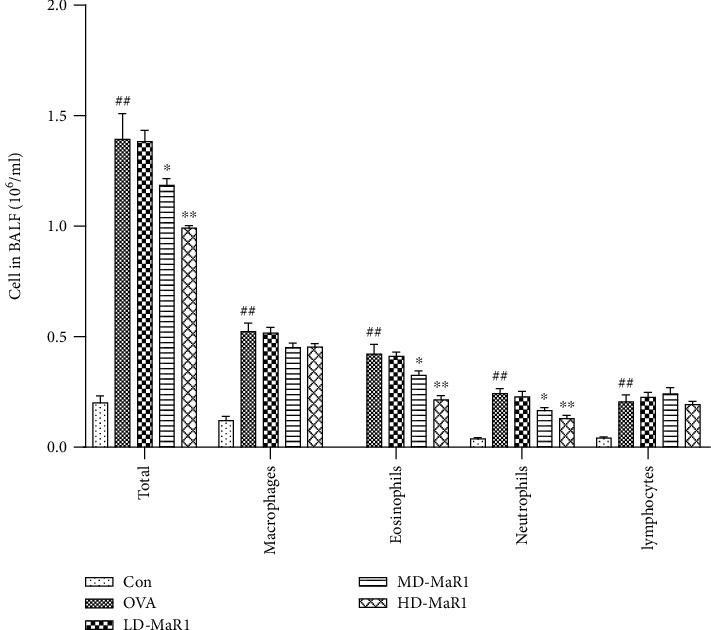
The experimental mice were subjected to alveolar lavage 24 hours after the last OVA aerosol challenge, and the BALF was taken for total cell counting and classification by Wright's stain. MaR1 treatment suppressed OVA-induced inflammatory cell recruitment in a dose-dependent manner. Data expressed as mean ± SEM (*n* = 6/group). ^#^*P* < 0.05, ^##^*P* < 0.01 vs. the control group; ∗*P* < 0.05, ∗∗*P* < 0.01 vs. OVA.

The results obtained from the analysis of inflammatory cell recruitment in BALF are shown in Figure 3. In comparison with the control group, the OVA group showed a notably increased total number of cells, as well as increased numbers of infiltrating macrophages, eosinophils, neutrophils, and lymphocytes in the BALF (all *P* < 0.01). In addition, compared with the OVA group, the MaR1-treated groups showed an obvious dose-dependent decrease in total inflammatory cells, neutrophils, and eosinophils in BALF (*P* < 0.01 to *P* < 0.05). However, administration of MaR1 had no significant effect on macrophages and lymphocytes in the BALF.

### 3.3. MaR1 Administration Suppressed OVA-Induced Th2 Associated Cytokine Production in BALF

**Figure 4:**
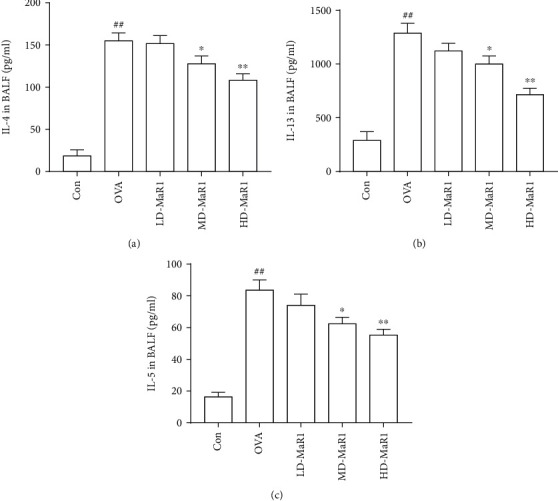
MaR1 treatment reduced OVA-induced Th2 cytokine levels in BALF. (a) IL-4 level in BALF. (b) IL-13 level in BALF. (c) IL-5 level in BALF. Data expressed as mean ± SEM (*n* = 6/group). ^#^*P* < 0.05, ^##^*P* < 0.01 vs. the control group; ∗*P* < 0.05, ∗∗*P* < 0.01 vs. OVA.

To analyze the effect of MaR1 on lung inflammation, the level of inflammatory cytokines was examined in BALF from mice administered a diverse range of concentrations of MaR1 (Figure 4). In this study, ELISAs were used to test the effect of MaR1 on OVA-induced Th2 cytokine production. The levels of the Th2-associated cytokines IL-4, IL-13, and IL-5 in the BALF of OVA-sensitized and challenged mice were significantly increased compared to those in the BALF of control mice (all *P* < 0.01). Compared with those in the OVA group, the levels of IL-4 (a), IL-13 (b), and IL-5 (c) were obviously downregulated in the high-dose MaR1 group (all *P* < 0.01) and medium-dose MaR1 group (all *P* < 0.05), whereas the low-dose MaR1 group showed no significant effect.

### 3.4. MaR1 Reduced the Level of IgE in the Serum

**Figure 5:**
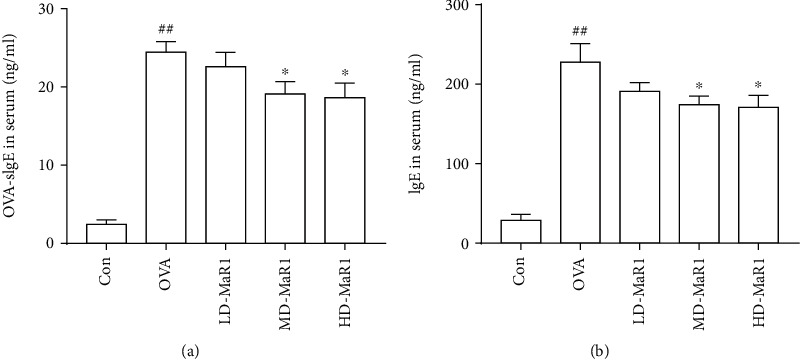
The mice were sacrificed 24 hours after the last OVA aerosol challenge; blood was taken by cardiac puncture and serum was collected. (a) OVA-specific IgE level in serum. (b) IgE level in serum. Data expressed as mean ± SEM (*n* = 6/group). ^#^*P* < 0.05, ^##^*P* < 0.01 vs. the control group; ∗*P* < 0.05, ∗∗*P* < 0.01 vs. OVA.

The total IgE (Figure 5(a)) and OVA-specific IgE (Figure 5(b)) levels in the OVA group were markedly upregulated compared to those in the control group (all *P* < 0.01). However, both the medium- and high-dose MaR1 treatments significantly reduced the total OVA and OVA-specific IgE levels in the serum (all *P* < 0.05); however, the high-dose treatment still performed better. Furthermore, the low-dose MaR1 group showed a slight decrease in OVA-specific IgE and total IgE, but the difference was not significant.

### 3.5. Effects of MaR1 on the Degradation of I*κ*B*α* and the Activation of NF-*κ*B in the Mouse Asthma Model

**Figure 6:**
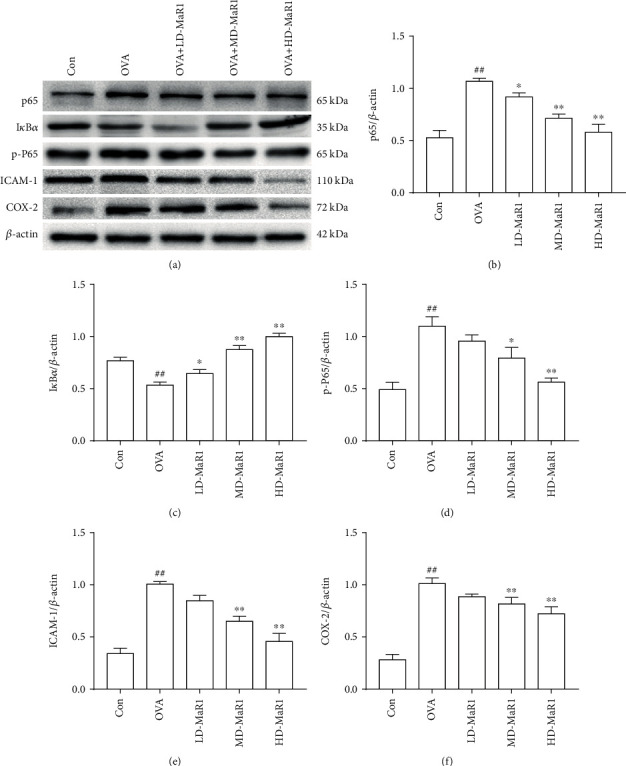
Effects of MaR1 on OVA-induced NF-*κ*B activation in lung tissue. (a) p65, I*κ*B*α*, p-P65, ICAM-1, and COX-2 protein expression representative Western blot images. (b–f) The relative density quantifications of p65, I*κ*B*α*, p-P65, ICAM-1, and COX-2. The results were expressed as the ratio of p65, I*κ*B*α*, p-P65, ICAM-1, and COX-2 relative to *β*-actin, respectively. Data expressed as mean ± SEM (*n* = 6/group). ^##^*P* < 0.01, ^#^*P* < 0.05 vs. the control group; ∗*P* < 0.05, ∗∗*P* < 0.01 vs. OVA.

An important role is played by NF-*κ*B in the pathogenesis of asthma. To verify the effect of MaR1 treatment on OVA-induced inflammation, we investigated the effect of MaR1 on I*κ*B*α*, an inhibitor of NF-*κ*B. Our results indicated that I*κ*B*α* degradation was increased, and the levels of p-P65 and p65 were increased in the OVA-induced asthmatic mice (all *P* < 0.01). As shown in Figure 6, the basal level of I*κ*B*α* (c) was high in the control group, whereas it was substantially decreased in the OVA-treated group and increased after MaR1 treatment (*P* < 0.01 to *P* < 0.05). Interestingly, with the addition of MaR1, the OVA-induced increase in p-P65 (d) and p65 (b) levels was downregulated in a dose-dependent manner (*P* < 0.01 to *P* < 0.05).

### 3.6. MaR1 Affects COX-2 and ICAM-1 Expression in the OVA-Induced Asthma Mouse Model

**Figure 7:**
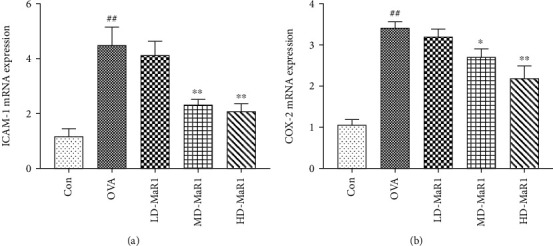
qRT-PCR assays were used to detect the expression levels of NF-*κ*B downstream inflammatory genes, such as ICAM-1 and COX-2. (a) The mRNA expression levels of ICAM-1. (b) The mRNA expression levels of COX-2. Data expressed as mean ± SEM (*n* = 6/group). ^##^*P* < 0.01, ^#^*P* < 0.05 vs. the control group; ∗*P* < 0.05, ∗∗*P* < 0.01 vs. OVA.

As shown in Figures 7(a) and 7(b), the mRNA expression levels of ICAM-1 and COX-2 were significantly enhanced by stimulation with OVA (all *P* < 0.01), while MaR1 treatment diminished the OVA-induced ICAM-1 and COX-2 mRNA expression in a dose-dependent manner (*P* < 0.01 to *P* < 0.05). Specifically, the high-dose MaR1 treatment significantly reduced the ICAM-1 and COX-2 mRNA expression levels in the OVA-induced asthma mice. Consistent with the mRNA expression of ICAM-1 and COX-2, both medium and high doses of MaR1 reduced ICAM-1 (Figure 6(e)) and COX-2 (Figure 6(f)) protein expression levels (all *P* <0.01). Notably, after treatment with MaR1, the protein expression of ICAM-1 and COX-2 was most significantly reduced at the high dose.

## 4. Discussion

Allergic asthma is a chronic inflammatory disease of the airway, the features of which are airway inflammation, increased IgE secretion, increased EOS, lymphocyte infiltration, and increased mucus secretion [25, 26]. These factors can directly or indirectly damage the airway structure, causing a large amount of shedding of the tracheal epithelial mucosa and mucus-induced embolisms. Our histopathological findings showed that MaR1 reduced inflammatory cell infiltration and airway goblet cell proliferation in lung tissue. Concomitantly, another important finding was that MaR1 caused an obvious decrease in the total number of inflammatory cells, eosinophils, and neutrophils in alveolar lavage fluid in a dose-dependent manner, which is consistent with previous Levy et al.'s studies [27]. However, in the present study, the inhibitory activity of MaR1 on OVA-induced macrophage and lymphocyte recruitment was not outstanding, which may be related to the anti-inflammatory properties of MaR1. Gong et al. pointed out in a study of acute lung injury that the inhibitory effect of MaR1 on macrophage and lymphocytes was not obvious, and even increased [23].

The pathogenesis of asthma is complex and not fully understood. Among the underlying pathologies, immune system abnormalities occupy an important position in the pathogenesis of bronchial asthma. Classical immunology states that a Th1/Th2 imbalance is a key factor in the pathogenesis of asthma and even airway inflammation [28]. Th1 cells are characterized by the secretion of IL-2 and IFN-*γ* and exert a cellular immune effect [29]. The activation of Th2 lymphocytes is a significant factor in the development of asthma, which can stimulate the synthesis of IgE and the infiltration of inflammatory cells by releasing representative cytokines such as IL-13, IL-4, and IL-5. In addition, Gavet et al. [30] found that the lack of the key Th2 cytokines IL-4, IL-5, or IL-13 all had a significant diminution effect on the asthma features of mice in the OVA model. IgE is an important immunoglobulin that mediates the type I hypersensitivity reaction [31]. When an allergen is combined with IgE antibody, it induces mast cells to degranulate and release cytokines, which causes the occurrence and development of the disease. Therefore, the level of serum IgE is directly related to the severity of the clinical symptoms [32]. In the present study, the administration of MaR1 to asthmatic mice reduced the levels of Th2-related factors (IL-4, IL-13, and IL-5) in the BALF. Our results also show that MaR1 treatment obviously suppressed the levels of total IgE and OVA-specific IgE in the serum. Collectively, these results indicate that MaR1 reduces the Th2 immune response.

**Figure 8:**
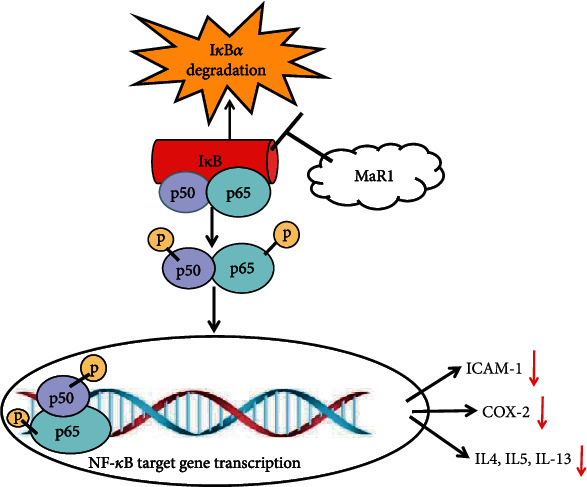
Model explaining the mechanism underlying the anti-inflammatory effects of MaR1. MaR1 treatment decreased the levels of IL-4, IL-5, and IL-13 and inhibited COX-2 and ICAM-1 expression via the suppression of NF-*κ*B signaling in the OVA-induced asthma mouse model. MaR1 is a potential anti-inflammatory compound that could ameliorate inflammation in the OVA-induced asthma mouse model.

Nuclear factor-*κ*B (NF-*κ*B) is a transcription factor that exists in a variety of cells and plays an essential role in the gene transcription of a variety of inflammatory factors, such as COX-2, ICAM-1, IL-8, and TNF-a [6, 7]. With the degradation of the inhibitory protein I*κ*B*α* by the proteasome, the NF-*κ*B signaling pathway is activated and then exerts a proinflammatory effect by regulating the expression of downstream genes such as proinflammatory cytokines [33, 34]. Therefore, regulating these pathways to manage asthma and reverse asthma inflammation and airway remodeling has become a very attractive strategy [35]. This study found that the degradation of I*κ*B*α* was significantly increased in the presence of OVA and that MaR1 treatment inhibited I*κ*B*α* degradation in a dose-dependent manner. The protein levels of p-P65 and p65 in the asthma model group were higher than those in the control group [36], suggesting that p-P65 and p65 may be involved in the development and progression of asthma. Furthermore, with the addition of MaR1, the OVA-induced increase in p-P65 and p65 levels was downregulated in a dose-dependent manner, indicating that MaR1 treatment inhibited NF-*κ*B activation. MaR1 regulates p-P65 and attenuates airway inflammation in asthmatic mice, probably because NF-*κ*B-dependent gene transcription is inhibited and inflammatory factor production is blocked, as shown in Figure 8.

COX-2 is an essential rate-limiting enzyme involved in prostaglandin synthesis. Arachidonic acid is metabolized into various PGI products, which can induce various physiological effects, including airway spasm and airway hyperresponsiveness, and thus induce acute asthma attacks. Our experimental results show that MaR1 treatment inhibits the expression of COX-2 in lung tissue. MaR1 treatment reduces the expression of COX-2, which further reduces the production of prostaglandin E2, an important mast cell mediator and strong immunomodulator that promotes fever, swelling, pain, and inflammation [37].

ICAM-1, namely CD54, is a transmembrane glycoprotein belonging to the immunoglobulin superfamily, which is widely distributed on the surface of antigen-presenting cells, granulocytes, dendritic cells, lymphocytes, epithelial cells, and other cells [38, 39]. ICAM-1 is expressed at low levels on resting endothelial cells, and ICAM-1 expression on the surface of various cells is increased in response to allergen stimulation [40]. Increased ICAM-1 expression can increase the susceptibility to inflammation. In airway inflammation, ICAM-1 in epithelial cells and endothelial cells can induce inflammatory cell recruitment from the blood to the site of inflammation and play a key role in adhesion and migration [41]. In asthma, ICAM-1 mainly regulates the adhesion between cells and increases adhesion between inflammatory cells and airway epithelial cells [42, 43]. Herein, we found that ICAM-1 expression in lung tissue is reduced in MaR1-treated asthmatic mice. According to the above results, MaR1 may reduce the level of ICAM-1 reverse transcription, thereby reducing the synthesis of ICAM-1 protein and ultimately reducing the adhesion of inflammatory cells, mainly EOs, to the airway epithelium.

## 5. Conclusion

Our study indicates that MaR1 plays an anti-inflammatory role in OVA-induced asthma inflammation by suppressing the degradation of I*κ*B*α* and the NF-*κ*B signaling pathway, strongly suggesting that MaR1 may be used as a potential drug for the treatment of asthma in the future.

## Figures and Tables

**Table 1:** Primer sequences and length of the amplified product.

Primer name		Primer sequence (5′-3 ′)	Product length
ICAM-1	Forward primer	TCCATCCATCCCAGAGAAGC	201 bp
	Reverse primer	GCCACAGTTCTCAAAGCACA	
COX-2	Forward primer	CCGTGGGGAATGTATGAGCA	168 bp
	Reverse primer	GGGTGGGCTTCAGCAGTAAT	
*β*-Actin	Forward primer	GTGGGAATGGGTCAGAAGGA	226 bp
	Reverse primer	TCATCTTTTCACGGTTGGCC	

## Data Availability

The provided data supporting the findings of this study are included within the article.
